# Unintentional Activation of Translation Equivalents in Bilinguals Leads to Attention Capture in a Cross-Modal Visual Task

**DOI:** 10.1371/journal.pone.0120131

**Published:** 2015-03-16

**Authors:** Niharika Singh, Ramesh Kumar Mishra

**Affiliations:** 1 Centre of Behavioural and Cognitive Sciences, University of Allahabad, Allahabad, India; 2 Centre for Neural and Cognitive Sciences, University of Hyderabad, Hyderabad, India; University of Akron, UNITED STATES

## Abstract

Using a variant of the visual world eye tracking paradigm, we examined if language non- selective activation of translation equivalents leads to attention capture and distraction in a visual task in bilinguals. High and low proficient Hindi-English speaking bilinguals were instructed to programme a saccade towards a line drawing which changed colour among other distractor objects. A spoken word, irrelevant to the main task, was presented before the colour change. On critical trials, one of the line drawings was a phonologically related word of the translation equivalent of the spoken word. Results showed that saccade latency was significantly higher towards the target in the presence of this cross-linguistic translation competitor compared to when the display contained completely unrelated objects. Participants were also slower when the display contained the referent of the spoken word among the distractors. However, the bilingual groups did not differ with regard to the interference effect observed. These findings suggest that spoken words activates translation equivalent which bias attention leading to interference in goal directed action in the visual domain.

## Introduction

It is well known that bilinguals activate conceptual as well as lexical representations from multiple lexicons during speaking, listening, and reading words in any one language [1–10]. Several recent studies have further shown that bilinguals spontaneously access translations of words automatically when these are not task relevant in monolingual contexts [11–13]. Therefore, it is logical to assume that bilinguals may face some interference in a primary task due to spurious language non-selective activation of lexicon where such activations are not task relevant. Earlier studies have found bilinguals to be slow in picture naming [14–15] and tasks that require single lexical decision [16–17] indicating parallel language activation interfering with speech production. However, from these studies, it is not clear if such automatic activation of lexicon can also interfere with a task which is non-linguistic i.e. launching a saccade towards a visual target. There have been a few studies showing interference in cross-modal situations, particularly when selective attention has to be paid to non-linguistic visual information [18]. In this study, we examined if automatic activation of translation equivalents leads to attention capture and affects oculomotor programming in bilinguals in a task that called for attention to visual colour change of line drawings. We used both high and low proficient Hindi-English bi-scriptal bilinguals who differed in their L2 proficiency to give the study a developmental perspective. Very little is currently known about the interfering effects of spoken words on visual processing in bilinguals. Bilinguals are a good candidate to study this since they have been known to spontaneously activate linguistic information from the unused language in different modalities.

### Translation activation and interference in bilinguals

In this study, we exploit the fact that bilinguals spontaneously activate translation equivalents during spoken word processing [19]. We tested if bilinguals will show interference in a visual task when a task irrelevant spoken word leads to the activation of translation equivalents which in turn affect looks towards distracters matching these activations. As for the parallel activation of languages seen in bilinguals there have been two types of studies. One group of studies has explored activation of words that share phonology in two languages [7, 9, 12, 20–21]. Others have found evidence for automatic activation of translations during bilingual language processing using ERP or RT based methods [5, 22–23]. Zhang, Van Heuven and Conklin (2011) observed that Chinese-English bilinguals spontaneously translated English words into Chinese words and further decomposed the morphemes in a lexical decision task [24]. It has been also shown that both high and low proficiency bilinguals activate task irrelevant translation equivalents during spoken word comprehension [12].

Consolidating the cross-modal nature of findings from visual world type studies on bilingualism and non-selective lexical activation, Shook & Marian (2013) [19] in their BLINCS model predict cross language activation for both same script and different script bilinguals. Further, the authors show that listening to a word leads to the activation of its translation equivalent in bilinguals. For example, with the Spanish word ‘ tenedor’ as input, the English translation equivalent ‘fork’ was active apart from within language and cross-language onset competitors. A feedback mechanism operating from the semantic level to the phonological level leads to translation activation. Give the English target ‘face’, higher activations were recorded for the translation equivalent ‘cara’ in Spanish as well as a phonological competitor ‘cama’ [19]. The model assumes lateral mapping which leads to spreading of activation from within language competitors to cross language competitors.

Activation of words that are phonologically related to the translation equivalents suggests ‘lemma’ level connections between words in the bilingual’s two different lexicons [25]. Therefore, these connections are much more conceptually mediated than superficial lexical level connections. Translation priming studies with bilinguals of different levels of proficiencies have shown that the magnitude and direction (L1-L2 and L2-L1) of translation differ [2, 7, 10, 26–29]. Bilinguals have been shown to activate translation equivalents when the task is non-linguistic in nature automatically [30]. Wu, Cristino, Leek & Thierry (2013) presented geometrical shapes among distractor English words to Chinese-English bilinguals in a visual search task and measured eye movements. Results showed that participants fixated more on a word whose Chinese translation overlapped phonologically with the Chinese word for circle or square. The authors claimed that Chinese-English bilinguals accessed translations in L1 even when the task is entirely non-linguistic. This evidence suggests that bilinguals do face some interference in a task even though the response required is non-linguistic [30].

We took two groups of bilinguals who differed with regard to their L2 proficiency. Blumenfeld & Marian (2013) have shown that high low proficient bilinguals differ in the time course of parallel language activation [31]. The Revised Hierarchical Model (RHM) explicitly links translation to L2 proficiency [32–33]. According to the RHM, high proficient bilinguals need not translate words, since they can directly access the meanings of L2 words, but low proficient bilinguals use the translation route. However, previous studies show that even highly proficient bilinguals who lived in L2 dominant environment show interference in a translation recognition task [11]. Similar results have been obtained with high proficient bilinguals who live in a L1 dominant language environment [12]. Therefore, both high and low proficient bilinguals activate translations of words unintentionally. We assumed that if that is the case, then these bilinguals should look towards the phonological cohort of the translation equivalent with the onset of the spoken word and therefore will be delayed in programming a saccade towards the target picture which changed colour. They should also be slower when the display contains the direct visual referent of the spoken word.

### Spoken words and attention capture

In this study, we examined if being a bilingual leads to interference in a visual task. The connection between bilingual lexical access and visual information processing is explained by models like BLINC [19] that exclusively consider visual world eye tracking data. Bilinguals activate conceptual information in both of their languages when they merely see pictures without any linguistic input [34]. In the paradigm used in this study, interference from the spoken words will only interfere with visual processing, if bilinguals activate phonological and conceptual information from line drawings automatically, even though the task requires attention to be focused on the colour change. Therefore, it is important to examine if bilinguals suffer interference in the visual modality because of spoken word input that leads to cross-language activation. While earlier studies have shown interference in bilinguals because of automatic access of translations, they have not examined the cross-modal nature of such interference [22–23]. Spoken words are hard to ignore and they hold attention, thus distracting from a primary task. Spoken words cause attention bias towards irrelevant things in the environment. Irrelevant speech has been shown to induce interference in memory and attention tasks, suggesting automatic processing [35–36]. These data suggest that spoken words directly engage the attention system, thus diverting intentional goal plans since one can access semantics from them rather automatically. Studies with cross-modal stimuli have shown that attention capture through irrelevant spoken words is automatic in the sense that participants are unable to ignore these task irrelevant stimuli [18, 37]. Some other studies have shown that spoken words can have a facilitative effect on visual perception [38]. Similarly, emotional content of words have been shown to capture attention [39]. Therefore, it is important to understand the mechanisms of interference produced by spoken words in the bilingual context and particularly in the visual domain when they are irrelevant to the task at hand.

In this study, we use the visual world eye tracking paradigm which offers a continuous measure of online processing during spoken word processing. The paradigm is suitable for studying mechanisms related to attention, automaticity and control [40]. The visual world eye tracking paradigm offers an excellent avenue to study subtle changes in eye movements and attention states during simultaneous processing of both visual and spoken information (see [6, 41–43] for reviews). In this paradigm eye movements to line drawings are tracked while participants listen to spoken words. Crucially, one of the line drawings happens to be related to the spoken words on some psycholinguistic features, i.e., phonological cohort, semantic, colour etc. Visual world studies show that listeners activate both phonological and semantic information during listening to spoken words and look at visual objects that matched with this representation [44–45]. Listening to a spoken word at once leads to activation of phonology as well as semantics. It appears that such activations are spontaneous and engage the oculomotor system. Salverda & Altmann (2011) observed that listeners are slow in a visual target detection task when irrelevant spoken words are presented. In this study saccades were slower towards target pictures when spoken words referred to distracters present in the scene [18]. Lupyan & Swingley (2011) asked participants to name objects during a visual search task. It was found that search was more efficient when the spoken verbal label matched the search target and there was higher interference when the name was different from the search target [46]. This indicates that spoken words processed unintentionally narrow down the search when the extracted semantics matches with the search target. Visual referents that are related to the spoken words compete with the target for selection and bias attention causing interference. Cleland, Tamminen, Quinlan, & Gaskell (2012) asked participants to make a response to a visual stimulus (a word, a face or a shape) in the presence of a spoken word. Response times were higher when the spoken word's uniqueness point was closer to the appearance of the visual stimulus and also effects were stronger as the gap between the spoken word and the visual stimulus decreased [47]. Further, response interference was higher when the visual stimulus was a word compared to a shape and a picture. These data suggest that spoken words automatically receive rich phonological as well as semantic processing and thus create attentional bottleneck during the search for visual targets. Even the unintentional processing of spoken words can lead to shifts in attentional states which in turn may lead to changes in goal planning and action selection.

### The current study

From the past studies, it is not clear if automatic semantic/conceptual access during unintentional processing of spoken words leads to distraction in a visual task in bilinguals. Further, past studies have not shown if automatic translation is contingent upon one's language proficiency, particularly, in bi-scriptal bilinguals that live and work in L1 dominant contexts, as in India. Therefore, we choose to exploit the bilingual attribute of parallel lexical activation to show its automaticity as well as to show interference during an audio-visual task. In the present study, we had two goals. First, to examine language non-selective activation of translation equivalent in bilinguals when an ocular response is sought to a visual task. Secondly, to examine if language proficiency has any effect on the observed interference given the fact that proficiency has been linked to parallel language activation. Earlier reports suggest that higher the language proficiency, greater is the magnitude of parallel language activation [5, 8, 11, 21]. Therefore, we were curious if such bilinguals with high L2 proficiency will show higher interference in a visual task. The use of bilinguals who use two languages with different scripts was considered as a novel component. Since, there has not been much work showing cross-modal activation of translation in bi-scriptal bilinguals. Participants looked at a visual array of four line drawings and were asked to make an eye movement towards an object that turned green after a certain interval. This was a purely visual task which required no language processing. We adopted the paradigm from Salverda and Altman (2011) to use it in a bilingual setting since it can help track implicit activations of cross-language competitors[36]. Crucially, on experimental trials, the display contained a picture whose name was a phonological competitor of the translation equivalent. For example, if the spoken word was ' gun', the display contained a picture of 'bandar' (Monkey) which was the phonological neighbour of the translation equivalent' bandook' (Gun). On some of the trials, the spoken word had a direct referent in the display. Participants were told that the spoken words are irrelevant to the visual search task. We expected distraction on those trials where the display contained the referent of the spoken words following Salverda & Altmann (2011) [18]. We further expected that the Hindi-English bilinguals would spontaneously access the translation equivalents of L2 words, and this may cause eye movements towards objects whose names overlap. It was predicted that interference will be seen only if translation is automatic in bilinguals and there should be no distraction on trials where the spoken word had no match of any type on the display. If participants do not access semantics automatically from spoken words, then we should not see interference in the translation and the referent present conditions. We measured the saccadic latency towards the target object (the object which changed colour) and also the proportion of fixations to different objects after the spoken word offset. In sum, we used the visual world eye tracking paradigm to examine interference caused by spoken stimuli in a visual task.

## Material and Methods

### Participants

Two groups of bilinguals were recruited for the study with twenty six participants in each group. The first group consisted of 26 high proficient Hindi-English bilinguals (Mean age = 21.7, *SD* = 2.8) from the Allahabad University. The second group consisted of twenty six low proficient Hindi-English bilinguals (Mean age = 20.3, *SD* = 2.2). Allahabad is a Hindi dominant area located in the Indian province of Uttar Pradesh. Participants were native speakers of Hindi (L1) and had formally acquired English at school as their L2. Proficiency screening measures were administered to all the participants (Table 1 & 2). This self-devised language proficiency background questionnaire required the participants to indicate their age of acquisition, percentage of time exposed to L1 and L2, language preference etc (Table 2). It also included questions related to proficiency for both L1 and L2 in speaking, reading, writing and understanding on 5 point *Likert* scale (Table 1).

**Table 1:** Self-ratings for reading, writing, speaking and comprehension in L1 and L2. Standard deviations are given in parentheses.

	Speaking		Listening		Reading		Writing	
	L1	L2	L1	L2	L1	L2	L1	L2
High Proficient Bilinguals	4.8(.32)	3.8(.65)	4.7(.53)	4.1(.54)	4.6(.54)	4.2(.42)	4.5(.64)	4.0(.56)
Low Proficient Bilinguals	4.7(.45)	1.9(1.0)**	4.5(.50)**	2.1(.69)**	4.6(.47)**	2.5(1.2)**	4.4(.57)**	2.1(1.3)**

**p<.*001*

Self-ratings scale: 1 = poor, 2 = functional, 3 = Fair, 4 = Good, 5 = very good

*Note*: L1 = Hindi. L2 = English.

**Table 2:** Comprehension passage and Lextale scores for the participants of both the groups.

	High Proficient Bilinguals	Low Proficient Bilinguals
Mean age of acquisition of L1	3.5(3.5)	4.1(.93)
Mean age of acquisition of L2	3.5 66)	8.1(3.3)
Percentage exposure to L1	2.9(.19	3.0(0.0)
Percentage exposure to L2	2.9(.27)	1.4(.50)**
Hindi passage score (out of 5)	4.6(.54)	4.4(.50)
English passage score (out of 5)	4.1(.65)	1.6(.97)**
Lextale Scores	83.9(5.3)	55.2(6.4)**

**p<.*001*

Apart from the subjective rating of proficiency, we also administered an objective L2 proficiency test called Lextale. Lextale [48] is a lexical decision task which consists of 60 words and non-words in English. It requires participants to respond to a string of letters by clicking on ‘yes’ option in case it is a meaningful word in English and clicking ‘no’ when it is a non-word. The low proficient bilinguals showed a significantly lower Lextale score as well as comprehension score in English as compared to the High proficient bilinguals (Table 2).

### Ethics statement

The study was approved by the ethics committee of the Allahabad University. All the participants of the study gave written informed consent prior to data collection and they were informed about the experimental protocol. The researcher who collected data also signed each consent form. They were also told that privacy as per law would be maintained with regard to their data.

### Stimuli

The stimuli consisted of 135 visual displays measuring 1024x768 pixels in resolution. Each visual display had four line drawings of common objects around a central fixation which was a small solid circle. Line drawings measuring 300x300 pixels were positioned at equal distance (8.5^0^) from the central fixation. Out of four, one of the line drawings was the target which changed its colour to green to which participants were required to make a saccade while other three were distractors. The target was always unrelated to the spoken word. The presentation of each display was paired with an auditory word in English. Three experimental conditions were created by manipulating the relationship between the spoken word and the distractors present in the display: i) Translation equivalent cohort condition: when the cohort of the spoken word’s translation equivalent (TE) was present as a competitor (ex. If the spoken word was “ring” (*angoothi*), then “*angoor*(Grapes)*”* as TE cohort was present as a competitor along with two distractors that were completely unrelated to the spoken input (See S1 Appendix); ii) Referent present condition: when the referent of the spoken input was present as a competitor among two distractors and the target (ex. If spoken word was “apple” then the line drawing of an apple was in the display as competitor)(See S2 Appendix), and iii) Control condition: when all the three distractors were unrelated to the spoken input(See S3 Appendix). The mean durations of spoken words in the translation cohort, referent present, and a control condition were 819ms (*SD* = 96.3), 776.7 ms (*SD* = 111. 9), and 745 ms (*SD* = 87.2) respectively. There were 45 trials in each condition, making total of 135 trials.

### Rating study

Ten proficient Hindi-English bilinguals who did not take part in the main experiment participated in the rating study. The participants were presented with a list of word pairs consisting of translation equivalents of the spoken word to be presented in the main experiment and a word which was phonologically similar (cohort competitor) to it. For example, if the spoken word was ‘lock’ then its translation equivalent ‘taala’ was presented with a phonologically similar word ‘tara (star) for rating. Care was taken to see that these were the only translations of the Hindi words. The participants were asked to judge the phonological similarity between the translation equivalents and the phonologically similar word on a 5 point scale (where *1* represented ‘highly dissimilar sounding” and *5* represented ‘highly similar sounding’). The mean phonological similarity between the translation equivalents (Hindi) of the English spoken word and the phonologically similar word was 3.9 (*SD* = 0.57).

Along with this, the same participants also did a translation agreement task which required them to write all the possible translations of the spoken word. This was to ensure that each spoken word had only one unique translation. The participants showed mean percentage translation agreement of 96.8% (*SD* = 6.6).

Apart from the subjective phonological ratings we also measured the phonological distance and similarity between the translation equivalent (of the spoken target) and the cohort competitor words and the unrelated distractors using the Levenshtein’s distance scale. This measures the minimum number of changes that are required to get an output string of letters from one input. The mean phonological distance between translation equivalents of English spoken words and their cohort competitors was significantly lower (*M* = 2.2, *SD* = .84) than the phonological distance between the translation and the target (M = 5.9, *SD* = 1.5), *t* (44) = -14.7, p = .001, and the unrelated distractors (*M* = 7.6, *SD* = 1.2), *t* (44) = -21.0, *p* = .001. Care was taken to see that distractors did not resemble spoken words [49] in any manner.

We also obtained ratings for the phonological and semantic similarity between spoken word and the visual target items, translation cohort competitors, and unrelated distractors from the same participants on the 5 point Likert scale. It was to ensure that spoken word did not match the target or the translation cohort competitors at phonological or semantic level. A repeated measure ANOVA was conducted on the phonological similarity ratings for the targets in the three conditions (i.e., translation equivalent cohort competitor, referent present, and control condition). The main effect of condition was not significant, *F*(2, 88) = .47, *p* = .62; showing low phonological similarity ratings for target in translation cohort competitor condition(1.01, *SE* = .01), referent present condition (1.02, *SE* = .02), and control condition (1.00, *SE* = .00). Similarly, ANOVA on semantic similarity was performed. The main effect of condition was not significant, *F*(2, 88) = 2.5, *p* = .08, showing a very low semantic similarity between spoken word and the targets in the translation cohort condition (1.12, *SE* = .04), referent condition (1.12, *SE* = .06), and unrelated condition (1.00, *SE* = .00).

Similarly, a repeated measure ANOVA on the phonological rating between the spoken words and competitors (i.e., translation cohort competitor, referent competitor, and unrelated distractors) was also conducted. The main effect of competitor type was found significant, *F* (2, 88) = 44000.0, *p* = .001, showing high phonological similarity between spoken word and the referent competitor (4.8, *SD* = .34) which differed significantly from the phonological ratings for translation cohort competitors (1.0, *SD* = .15) and unrelated distractors (1.0, SD = .13). Similarly, the semantic similarity ratings between spoken word and competitors (translation cohort competitor, referent competitor, and unrelated distractors) showed a significant effect of competitors, *F* (2, 88) = 3765.0, *p* = .001. It revealed that the semantic similarity between spoken word and referent competitor was very high (5.00, *SD* = .00) which significantly differed from the semantic similarity ratings for translation cohort competitors (1.17, *SD* = .35) and unrelated distractors (1.0, *SD* = .25)

### Procedure

**Fig 1:**
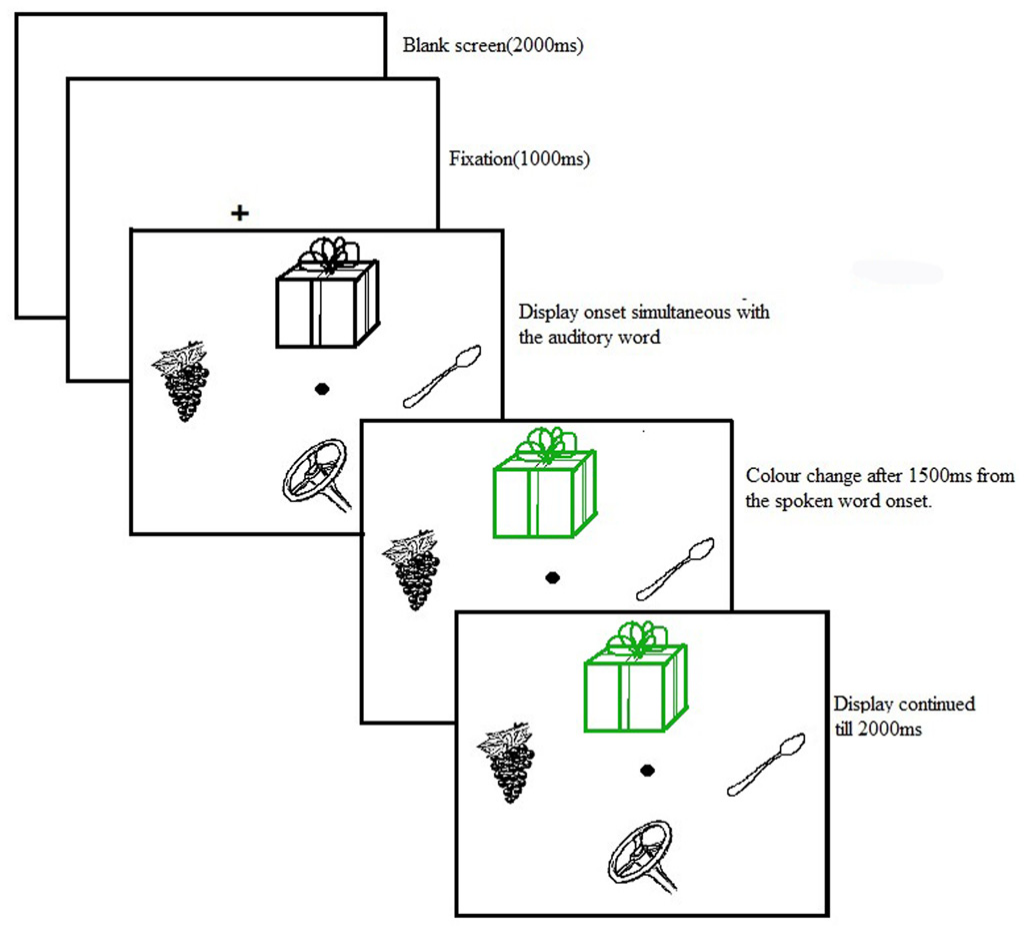
Sample trial sequence for TE cohort present condition where the spoken word was “Ring”(*angoothi*) which was paired with display containing translation cohort as competitor as *angoor*(Grapes).

Participants were naïve to the purpose of the experiments and no indication was given about the experiment being on bilingualism. Participants comfortably sat at a distance of 60 cm from a 17’ LCD colour monitor with 1024 X 768 pixel resolution. Eye movements were recorded with a SMI High speed eye—tracking system (Sensomotoric Instruments, Teltow) running with a sampling rate of 1250 Hz. In the beginning, participants were given a brief demo of a sample trial. Participants’ eye movements were calibrated at 13 different points at the beginning of the experiment. Each trial began with the presentation of a fixation cross (+) at the centre of the screen for 1000 ms. It was followed by the simultaneous presentation of a display containing four line drawings and a spoken word. After 1500 ms from the onset of the auditory word, one of the objects in the display changed its colour to green. The display remained on the screen till 2000 ms after the colour change. The participants were instructed to move their eyes towards the object that turned green in colour as soon possible. Participants were instructed not to move their eyes before the colour change and were required to fixate at the centre. Participants were informed that the spoken word was irrelevant to the task and they should make eye movements in response to the colour change and not to the spoken input. Soon after this, (on 40% of trials) participants were asked to give a response about the animacy of the target object (See Fig. 1). This was to make sure that participants attended to the visual object at all times. After this, a blank display was presented for 2000 ms and then the next trial began. Trials were randomised for each participant. The position of the target object on the screen with regard to the translation competitors and distracters were counterbalanced across trials for each participant. Thus, participants could not make an eye movement towards the target based on any strategy or remembered location from trial to trial. Spoken words were presented in the English language (L2) only and the experimenter explained the task instructions using English. It is important to note that the university setting in India is largely English based and thus using English should not explicitly raise the suspicion in the participants' mind that they were being tested for their bilingualism.

## Results

### Saccade latency

The data from each participant’s right eye were extracted using the Begaze analysis software (SMI, Teltow). Saccade latency to the target was calculated measuring the time lag between the colour change and the first correct saccade made towards it. Saccade latencies less than 80 ms (anticipatory) and higher than 1000 ms were excluded from further analysis (7.6%, SD = 5.6) of the trials for high proficient group and 9.5%, SD = 7.7) for low proficient group). We only analysed those trials where participants responded correctly by making an appropriate eye movement towards the target. Due to phonological similarity between the spoken word and the items in the visual display four trials from the Translation cohort condition were excluded from the final analysis.

**Fig 2:**
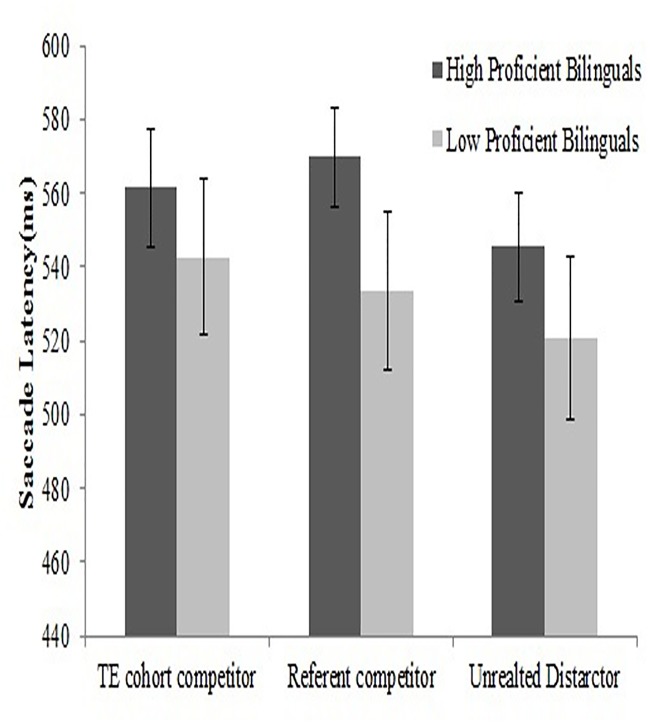
Showing mean saccade latencies to the target in the presence of referent competitor, TE cohort competitor, and unrelated distractor conditions.

A repeated measure of ANOVA, both subject-wise and item-wise were conducted with Distractor type (Referent competitor, Translation Cohort competitor, Unrelated distractor) as a within subjects factor and Group type (High proficient bilinguals vs. Low proficient bilinguals) as a between subject factor on saccade latency. The main effect of group was not significant, *F*_*1*_(1,50) = 1.13, p = .29, η2p = .02; *F*_*2*_ (1,79) = .23, *p* = .63. However, The main effect of distractor type was found highly significant, *F*_*1*_ (2,100) = 5.4, *p* = .006, η^2^_p_ = .09; *F*_*2*_ (2,158) = 4.17, *p* = .01, showing participants were significantly slower at launching a saccade towards the target in the referent competitor condition (551.59 ms, *SE* = 12.9) and the translation cohort competitor condition (552.16ms, *SE* = 13.2) than in the condition when the target was accompanied by unrelated distractors (533.14ms, *SE* = 13.2)(See S1 Dataset). The interaction between group and distractor type was not found to be significant, *F1* (2,100) = .93, *p* = .39, η^2^_p_ = .01; *F*_*2*_ (2,158) = .12, *p* = .88(See Fig. 2).

### Analysis of errors

First saccade landing on any object other than target after colour change was considered an error. The main effect of distractor type was significant, *F* (2,100) = 12.0, *p* = .001, η^2^_p_ = .19, indicating lower number of errors in the Translation cohort condition (14.0, *SE* = 1.1) which significantly differed from the number of errors in the referent present condition (16.7, *SE* = 1.2)and the unrelated distracter condition (15.7, *SE* = 1.0). The main effect of group was not significant, *F* (1, 50) = .39, *p* = .53, η^2^_p_ = .00; showing comparable errors for the high proficient bilinguals (14.8, *SE* = 1.6) and low proficient (16.2, *SE* = 1.6) bilinguals. There was no interaction of group with the distractor type, *F* (2, 100) = .09, *p* = .91, η^2^_p_ = .002.

### Fixation Proportions

Fixation proportions to TE cohort, referent competitor and distractors were calculated from the onset of the spoken word. To see how the fixation proportions gradually increased/decreased over time to each object we calculated fixation proportions for two time windows i.e. 0–300ms and 300–800ms. We considered the 0–300 as an early time-window and 300–800 as the late time window (see Blumenfeld& Marian, 2013). We did this analysis also to examine if t stronger and immediate activation of the translation equivalent will result into larger proportion of fixation towards the translation competitor in the early time window. It is then likely that the proportion of fixation towards translation competitors for the two groups of bilinguals should be different for these two time windows, if language proficiency influences activation of translation equivalents. Fixations to different objects were averaged for this analysis. A repeated measure of ANOVA, with distractor type (TE cohort competitor, Referent competitor, and distractor) as within subject factor and group as between subject factor, was conducted separately for the two time windows.

#### Time window 0–300ms after colour change

The main effect of distractor type on the fixation proportion was not found to be significant, *F* (2,100) = 1.0, *p* = .34, η^2^_p_ = .02; showing comparable fixation proportions for TE cohort competitors (33.2%, *SE* = .014), referent competitor (33.3%, *SE* = .011) and distractors (35.3%, *SE* = .007). The main effect of group was found to be significant, *F* (1, 50) = 4.5, *p* = .03, η^2^_p_ = .08; showing significantly higher fixation proportions for the low proficient bilinguals (35.1%, *SE* = .008) than the high proficient bilinguals (32.8%, *SE* = .008). The group type did not interact with the distractor type, *F* (2,100) = .42, *p* = .65, η^2^_p_ = .00.

#### Time window 300–800ms after colour change

This time window was selected since we expected translated related effects should show up in this time-scale [8]. The main effect of distractor type was not significant, *F*(2,100) = 1.0, *p* = .35, η^2^_p_ = .02; showing similar fixation proportions to TE cohort competitor(33.4%, *SE* = .01), referent competitor (32.9%, *SE* = .01) and distractor (35.2%, *SE* = .007). Even though the difference between overall fixation proportion for the high proficient bilinguals (32.7%, *SE* = .007) and the low proficient bilinguals (34.9%, *SE* = .007) was not much, nonetheless the main effect of group was found to be significant, *F* (1,50) = 4.9, *p* = .03, η^2^_p_ = .09. The group type did not interact with distractor type, *F* (2,100) = .25, *p* = .77, η^2^_p_ = .005

## Discussion

In this study we examined if bilinguals face interference in a visual task because of undesirable translation activation from task irrelevant spoken words. Both high and low proficient Hindi-English bilinguals were asked to program a saccade towards a line drawing that changed colour among other competitors. Both groups of bilinguals were slower in programming a saccade towards the target when the display either had a translation cohort or the direct referent of the spoken word as compared to when the display had completely unrelated objects. However, we did not observe any difference between the groups with regard to such interference. These results thus show that bilinguals activate translations of spoken words automatically and this can cause interference in a non-linguistic oculomotor task. In the Salverda & Altmann (2011) study, actual referents of spoken words were present in the display and this led to slower saccadic latencies to targets in an eye movement task in monolinguals [18]. This effect was interpreted in suggesting that spoken words can’t be ignored and listeners process their phonology and semantics unintentionally. We found both the groups showing higher latencies to the targets on conditions when the actual referent was present, replicating this effect. However, we also found higher latencies to targets when displays contained a phonological competitor of the spoken word’s translation for both the groups. Saccade latency in the translation cohort present condition would not have been higher if bilinguals would not have activated translations of spoken words.

The data suggest that for bilinguals, spoken words led to activation of translations and this can lead to attention capture in a task that calls for a non-linguistic response. Thus, our paradigm, which was different from earlier paradigms like translation recognition and priming, was suitable to elicit implicit activation of translation activation in a cross-modal situation. These results thus extend previous observations that show bilinguals access translation information unconsciously, which affects their performance in tasks where such activations are not necessary [5, 11–13]. Our results also show that translation activation is automatic to the extent that it happens even when bilinguals are given an explicit non-linguistic task. In our task, it is likely that participants activated the names of objects and processed the meanings of spoken words, even though they had to make a response to the colour change. These unintentional linguistic activations led to slowing down of goal directed saccades.

From visual world studies it is known that spoken words trigger eye movements towards goal relevant visual depictions within 100ms [50]. This means very rapidly spoken words lead to attention bias among competing visual stimuli that result in shifting of the eyes. In our case, spoken words lead to activation of task irrelevant distractors causing attentional capture by matching objects. Interestingly, the participants had a top down goal of observing colour change in one object and moving their eyes towards it. However, the tug of war between the distractor activation leading to attentional bias and the top down goal caused some delay. Our results nevertheless extend other findings that show spoken words capturing attention [46].

We compared two groups of bilinguals who differed with their L2 proficiency. The RHM model [32] would predict the activation of translations only for the low L2 proficiency bilinguals but not for the high proficient bilinguals, since the low proficient bilinguals need to translate the L2 words into L1 words for comprehension. Ideally, we should have observed higher interference in the low proficient bilinguals in the translation cohort condition than the high proficient bilinguals. We did not observe a significant group difference with regard to such interference. However, we found interference not only in the low proficient group but also in the high proficient group. Our result thus replicates earlier findings that show even highly proficient bilinguals show translation interference [11]. The low proficient bilinguals were individuals who were late learners but had sufficient knowledge of English to know names of simple objects used in the study. Further, our study shows that even different script bilinguals show language non-selective activation of translations cross modally which is predicted by bilingual models like BLINCS [19]. It is important to note that BLINCS model does not predict cross-language activation in bilinguals with regard to developmental variables like proficiency. Other studies that have looked at language unconscious translation in bilinguals but have not compared any groups [13].

It is also possible that activation of translation in bilinguals is dependent on the nature of the task and the paradigms. As is evident from the proportion of fixation analysis at two time-windows, group had an affect both on the early as well as late windows. These results thus show activation of translation equivalents in bilinguals that led to slowness in the visual colour change task. These results also extend previous findings with monolinguals showing spoken words capture attention and slow down the visual target search [18]. The results also extend the previous findings with bilinguals that show automatic activation of translation equivalents with a cross-modal task. This study also provides first evidence of spurious translation activation in bilinguals irrespective of proficiency under a selective attention task.

It is important to note that both these groups were bilinguals and therefore such contrasts are different in nature compared to studies that compare monolinguals vs. bilinguals. This type of comparison makes sense for the Indian context since there are not many monolinguals to compare with bilinguals (as has been with most western studies). Most educated Indians have good proficiency in English as a second language. However, we would like to note that language use in India, particularly the use of English is highly context dependent. For example, the use of English at work as opposed to home or even among peer members. Participants in our study were all university students and used English at work most often but used Hindi at home. We assume these factors can influence studies that test bilinguals for their cognitive control or even lexical activation. We predict if our study is replicated with a set of monolinguals, with no knowledge of English, then we should not see any interference at all.

Our study thus shows that spoken words do interfere with visual object processing since they immediately activate meaning leading to attention capture attention by objects that have lexical overlap with such meanings. Importantly, they influence attention bias and thus create some type of processing attentional bottleneck. The delay thus results in slowing down of goal directed action i.e. manual or eye movements. Our study also establishes that bilinguals activate cross-language semantics spontaneously during spoken language processing. Our results with Hindi and English (Languages that do not share orthography) make this finding even more interesting. Not many studies have shown spontaneous activation of translation equivalents during spoken word processing in bi-scriptal bilinguals. However, such activations are predicted by interactive activation models. Our data thus shows that irrespective of differences in orthography, bilinguals extract semantics from spoken words and activate related words in the non-target language. Earlier it has been shown that orthographic information influences spoken word processing in monolinguals [18]. However, not many bilingual visual world studies have examined these issues during parallel language activation.

We assume that the bilingual context and issues related to language dominance should influence such findings and future studies should test them. These results thus add another angle to the growing number of studies that have been exploring bilingual’s executive control with both linguistic and non-linguistic tasks and correlations between them. We would like to emphasise that our participants were young adults and this group has not been studied a lot in the bilingual cognitive control literature. To conclude, we showed that bilinguals access translation information spontaneously, thus extending recent findings with a visual-world study. Such activations further can interfere with any ongoing task. In this study, we have shown that task irrelevant spoken words caused higher interference in a saccade task, when they matched with visually presented objects either conceptually or phonologically.

## Supporting Information

S1 Appendix Appendix containing stims used in the translation cohort present condition. (DOC)

S2 Appendix Appendix containing stims used in the competitor present condition. (DOCX)

S3 Appendix Appendix containing stims used in the unrelated condition. (DOCX)

S1 Dataset Dataset for saccade latency and errors for high and low proficient Hindi-English bilinguals. (XLSX)
