# Supplementary material for: Unintentional Activation of Translation Equivalents in Bilinguals Leads to Attention Capture in a Cross-Modal Visual Task
Source: PLoS One. 2015 Mar 16;10(3):e0120131. doi: 10.1371/journal.pone.0120131 (PMC4361716; doi:10.1371/journal.pone.0120131)
Supplement: S1 Appendix — (DOC) [file pone.0120131.s001.doc]

S2 Appendix .

| **Auditory spoken word** | **Translation** | **Target** | **TE cohort competitor** | **Distractor 1** | **Distractor 2** |
| --- | --- | --- | --- | --- | --- |
| bamboo | baans | telescope | bansuri(Flute) | snowman | thermos |
| bangle | kangan | slingshot | kanghi(comb) | plier | peacock |
| bear | bhalu | ostrich | bhaala(spear) | skunk | letter |
| brinjal | baingan | rake | bail(bull) | eskimo | dustpan |
| bullet | goli | dolphin | gobhi(cauliflower) | comb | church |
| bush | jhadi | chest | jhanda(flag) | deck | dime |
| butter | makkhan | farm | makkhi(housefly) | genie | heels |
| chair | kursi | iron board | kurta( shirt) | giraffe | flashlight |
| dance | naach | fence | naak(nose) | globe | hose |
| donkey | gadha | log | gada(mace) | medal | nest |
| elephant | haathi | pinecone | haath( hand) | purse | rainbow |
| farmer | kisaan | rooster | kitaab(book) | sled | step |
| flag | jhanda | tractor | jhadu(broom) | spade | wagon |
| garden | baag | vest | baagh(tiger) | zipper | scale |
| hunger | bhhokh | rug | bhoot(ghost) | submarine | popcorn |
| gun | bandook | Jump rope | bandar(monkey) | dress | cross |
| bell | ghanti | Sled | ghada(pitcher) | fox | railtrack |
| kite | patang | basket | patta(leaf) | dragon | ashtray |
| lock | taala | belt | taara(star) | balloon | cast |
| lotus | kamal | anvil | kalam(pen) | dresser | mixer |
| monkey | bandar | fork | bandook(gun) | beard | necklace |
| palm | hatheli | window | hathoda(hammer) | stethoscope | moose |
| parrot | tota | bread | tope(cannon) | rhinoceros | soldier |
| pen | kalam | slipper | kamal(lotus) | tomato | wagon |
| pig | suwar | ball | suraj(sun) | toe | desert |
| potato | aaloo | wizard | aaj(fire) | shirt | bus |
| parrot | tota | accordion | tope(cannon) | bathtub | pitcher |
| rabbit | khargosh | box | kharbooj(musk melon) | nurse | towel |
| rain | barsaat | camera | bartan(utensil) | sewing machine | hair |
| rice | chawal | stem | chaaku(knife) | bowl | grasshopper |
| ring | angoothi | present | angoor(grapes) | spatula | steering |
| rose | gulaab | submarine | gudiya(doll) | shirt | yoyo |
| seed | beej | microphone | been(flute) | farm | pin |
| song | gaana | alligator | gaay(cow) | finger | can |
| spinach | palak | Nail file | payal(anklet) | deer | paint |
| dog | kutta | robot | kurta( shirt) | flower | housefly |
| star | taara | house | taala(lock) | well | seal |
| sugar | cheeni | cigarette | cheenti(ant) | rock | curtain |
| sun | suraj | tail | suwar(pig) | sword | wall |
| thumb | angootha | fork | angoor(grapes) | ladybug | chair |
| wood | lakadi | cheese | ladki(girl) | window | cabbage |
| coriander | dhaniya | roof | dhanush(bow) | chimney | mouse |
| moon | chand | hat | chaku(knife) | trophy | hippo |
| fire | aag | roof | aam(mango) | hinge | mouse |
| wire | taar | ladder | taala(lock) | celery | bucket |
