# Supplementary material for: Unintentional Activation of Translation Equivalents in Bilinguals Leads to Attention Capture in a Cross-Modal Visual Task
Source: PLoS One. 2015 Mar 16;10(3):e0120131. doi: 10.1371/journal.pone.0120131 (PMC4361716; doi:10.1371/journal.pone.0120131)
Supplement: S2 Appendix — (DOCX) [file pone.0120131.s002.docx]

S3 Appendix

| **Auditory spoken word** | **Target** | **Referent as competitor** | **Distractor 1** | **Distractor 2** |
| --- | --- | --- | --- | --- |
| apple | igloo | apple | gun | bamboo |
| arrow | king | arrow | kite | window |
| bird | umbrella | bird | rose | acorn |
| balloon | queen | balloon | heart | man |
| baby | iron | baby | lipstick | mask |
| bed | plate | bed | raccoon | cone |
| bread | teapot | bread | unicorn | radio |
| brush | tire | brush | watch | razor |
| camel | tape | camel | whistle | robot |
| candle | bug | candle | trumpet | slide |
| cat | swing | cat | cannon | button |
| chicken | skeleton | chicken | canoe | pen |
| coat | ant | coat | spider | alligator |
| guitar | wall | guitar | shovel | chain |
| hat | fork | hat | barrel | cheese |
| horn | knob | horn | spoon | carrot |
| nose | chair | nose | hen | umbrella |
| purse | pillar | purse | ear | feather |
| rocket | flute | rocket | gun | hat |
| sheep | kangaroo | sheep | ladle | letter |
| shell | match | shell | necklace | onion |
| stool | pear | stool | rock | screwdriver |
| duck | aeroplane | duck | panda | sausage |
| door | shark | door | penguin | tent |
| fan | shovel | fan | squirrel | violin |
| tie | pyramid | tie | wine | walrus |
| tree | wing | tree | pumpkin | acorn |
| pot | bear | pot | carpet | clock |
| cup | shell | cup | banjo | cigarette |
| finger | basket | finger | crib | rock |
| eyes | kite | eyes | bat | curtain |
| owl | robot | owl | teeth | mirror |
| tank | flower | tank | whale | bee |
| tail | housefly | tail | flute | crab |
| sword | earring | sword | grasshopper | dog |
| wall | egg | wall | shark | acorn |
| whale | airplane | whale | igloo | ladder |
| fish | lemon | fish | tie | man |
| insect | moon | insect | potato | phone |
| shoe | sheep | shoe | roof | house |
| glove | scorpion | glove | cake | well |
| sock | tractor | sock | mouse | seal |
| cap | fish | cap | brush | grape |
| nail | pant | nail | flower | wrench |
| hammer | trophy | hammer | zebra | butterfly |
|  |  |  |  |  |
