# Supplementary material for: Unintentional Activation of Translation Equivalents in Bilinguals Leads to Attention Capture in a Cross-Modal Visual Task
Source: PLoS One. 2015 Mar 16;10(3):e0120131. doi: 10.1371/journal.pone.0120131 (PMC4361716; doi:10.1371/journal.pone.0120131)
Supplement: S3 Appendix — (DOCX) [file pone.0120131.s003.docx]

S4 Appendix

| **Auditory spoken word** | **Target** | **Distractor 1** | **Distractor 2** | **Distractor 3** |
| --- | --- | --- | --- | --- |
| bucket | pig | tree | rose | man |
| needle | wig | watch | whistle | trumpet |
| crown | canoe | spider | shovel | barrel |
| throne | carrot | knob | spoon | horn |
| king | lipstick | raccoon | unicorn | watch |
| branch | tire | tape | bulb | swing |
| man | onion | screwdriver | sausage | tent |
| girl | knob | chair | pillar | flute |
| horse | feather | crown | letter | onion |
| panda | squirrel | wine | spider | carpet |
| lizard | harp | key | pumpkin | knot |
| bottle | orange | door | rose | paper |
| coal | pizza | jar | chimney | boat |
| brick | chain | radish | umbrella | skirt |
| bridge | wallet | wood | jacket | glasses |
| cloud | mask | watch | turkey | paintbrush |
| hill | lobster | Safety pin | mountain | swan |
| paper | scissors | ring | shell | table |
| bag | ruler | nose | mixer | lizard |
| bean | nail | gorilla | acorn | banjo |
| road | barrel | spoon | hen | ear |
| turtle | kite | robot | flower | housefly |
| vase | acorn | ladder | man | phone |
| wolf | nail | hat | flower | wrench |
| wheel | bamboo | window | cauliflower | man |
| kettle | wing | bear | shell | crib |
| corn | fly | wall | gun | crib |
| cow | bomb | horse | baby | iron |
| toffee | mop | Ice-cream | bucket | match |
| clip | pelican | raft | mosquito | syringe |
| watch | peas | train | raccoon | stamp |
| turkey | suitcase | pipe | sheep | bell |
| zebra | sweater | bat | walnut | salt |
| skirt | bone | well | piano | mug |
| shirt | hoof | dinosaur | coat | deck |
| pan | dolphin | eye | cross | ghost |
| owl | helicopter | phone | flower | leopard |
| ostrich | potato | roof | chimney | mouse |
| lion | mailbox | sweater | light switch | Palm tree |
| hook | piano | man | rocket | Rolling pin |
| glass | typewritten | wheat | tennis racket | phone |
| fox | windmill | ladder | man | house |
| feather | house | well | seal | grape |
| jar | throne | whip | rock | crib |
| grapes | shark | igloo | tie | potato |
